# Role of intraparotid node metastasis in mucoepidermoid carcinoma of the parotid gland

**DOI:** 10.1186/s12885-019-5637-x

**Published:** 2019-05-03

**Authors:** Xingyu Niu, Qigen Fang, Fei Liu

**Affiliations:** 1 Department of Oral Medicine, The First Affiliated hospital of Zhengzhou University, Zhengzhou, People’s Republic of China; 2 Department of Head Neck and Thyroid, Affiliated Cancer Hospital of Zhengzhou University, Henan Cancer Hospital, Zhengzhou, China

**Keywords:** Intraparotid node, Parotid cancer, Mucoepidermoid carcinoma, Recurrence, Prognosis

## Abstract

**Background:**

Prognostic factors for parotid mucoepidermoid carcinoma (MEC) usually include disease grade, tumor stage, node stage, perineural invasion, and lymphovascular invasion. But the role of intraparotid nodes (IPNs) remains unclear, therefore, the study aimed to analyze the significance of IPNs in predicting recurrence in parotid MEC.

**Methods:**

One hundred and ninety patients were included for analysis finally. Data regarding demography, pathological characteristics, IPN metastasis, TNM stage, follow up was collected and evaluated. The recurrence-free survival (RFS) was the main study endpoint.

**Results:**

A total of 47 (24.7%) patients had IPN metastasis, and the IPN metastasis was significantly related to tumor stage, pathologic N stage, lymph-vascular invasion, perineural invasion, and disease grade. Recurrence occurred in 34 (17.9%) patients. For patients without IPN metastasis, the 10-year RFS rate was 88%, for patients with IPN metastasis, the 10-year RFS rate was 54%, the difference was significant (*p* < 0.001). Further Cox model analysis confirmed the independence of IPN metastasis in predicting the prognosis.

**Conclusion:**

The IPN metastasis is relatively common in parotid MEC, it is significantly related to tumor stage and disease grade, IPN metastasis means worse recurrence-free survival.

## Background

Parotid gland cancer is an uncommon neoplasm [1], but according to their architectural and cytological features, there were 24 types of malignant tumors classified by The World Health Organization [2, 3], mucoepidermoid carcinoma (MEC) is the most common pathologic subtype, some clinic-pathologic variables, including histologic grade, lymphovascular and perineural invasion as well as tumor stage, have been well described to be associated with the prognosis in parotid MEC [4–6].

Because of the exceptional growth process, there are intraparotid nodes (IPNs) in the parotid gland [7], its prognostic value remains unclear [8–11]. The IPNs are classified into superficial and deep lobe nodes [12], therefore the IPNs are significantly affected by different surgical procedures, compared to total parotidectomy, lateral or superficial parotidectomy has a lower probability to find all relevant IPNs, but in the above-mentioned researches [8–11], a certain number of procedures of lateral or superficial parotidectomy are included, therefore the exact IPN metastasis rate as well as its survival impact in patients with MEC remains unclear.

Therefore, the current study aimed to evaluate the IPN metastasis rate and the significance of IPN metastasis in predicting recurrence in parotid MEC.

## Methods

Our study was approved by the Zhengzhou University institutional research committee, and an informed consent agreement for medical research before initial treatment was achieved for all participants, and all experiments were performed in accordance with the Declaration of Helsinki.

Patients undergoing surgical treatment for primary parotid MEC during January 2001 and December 2017 were retrospectively tracked. Data regarding demography, pathological characteristics, TNM stage, IPN metastasis, and follow-up information was collected and assessed. The tumor stage and tumor grade were formulated based on the AJCC^8th^ classification and the WHO 2017 classification, respectively. Ultrasound and CT or MRI examination was conducted for every patient, and total parotidectomy was conducted when malignant epithelial disease was supported by frozen section of the primary tumor.

The relationship between the clinical-pathologic variables and the IPN metastasis was assessed by the Chi-square test. The main study endpoint was the recurrence-free survival (RFS), and it was calculated by the Kaplan-Meier method (Log-rank test), the Cox proportional hazards method was used to analyze the factors which were significant in the univariable analysis to determine the independent risk factors for the RFS. All statistical analyses were performed by SPSS 20.0, and a *p* < 0.05 was considered to be significant.

## Results

One hundred and ninety patients (101 female and 89 male) were included for analysis, the mean age was 48.7 years with a range from 18 to 85. Pathologic tumor stages of T1, T2, T3, and T4 presented in 27, 95, 45, and 23 cases, respectively. Neck metastatic node disease was found in 41 cases of the 62 patients undergoing a neck dissection. Negative (R0) and positive margin (R1/2) was achieved in 181 and 9 patients, respectively. Perineural and lymphovascular invasion was noted in 23 and 22 patients, respectively. Low grade MEC reported in 104 cases, intermediate grade in 62 cases, and high grade in 24 cases, .

A total of 47 (24.7%) patients had IPN metastasis. As described in Table 1, pathologic N stage, tumor stage, perineural invasion, lymph-vascular invasion, and disease grade were significantly related to the IPN metastasis (all *p* < 0.05).

**Table 1:** Association between intraparotid node metastasis and clinical-pathologic variables

Variables	Intraparotid node metastasis		p
	Positive(*n*=47)	Negative (*n*=143)	
Age			
≤ 50	20	72	
>50	27	71	0.353
Sex			
Female	29	72	
Male	18	71	0.176
Tumor stage			
T1 + T2	23	99	
T3 + T4	24	44	0.012
p-Neck stage *			
N0	6	15	
N+	30	11	0.001
Perineural invasion			
Yes	11	12	
No	36	131	0.006
Lymphovascular invasion			
Yes	11	11	
No	36	132	0.003
Disease grade			
Low	12	92	
Intermediate	24	38	
High	11	13	< 0.001

*: Patients undergoing neck dissection were analyzed

**Fig. 1:**
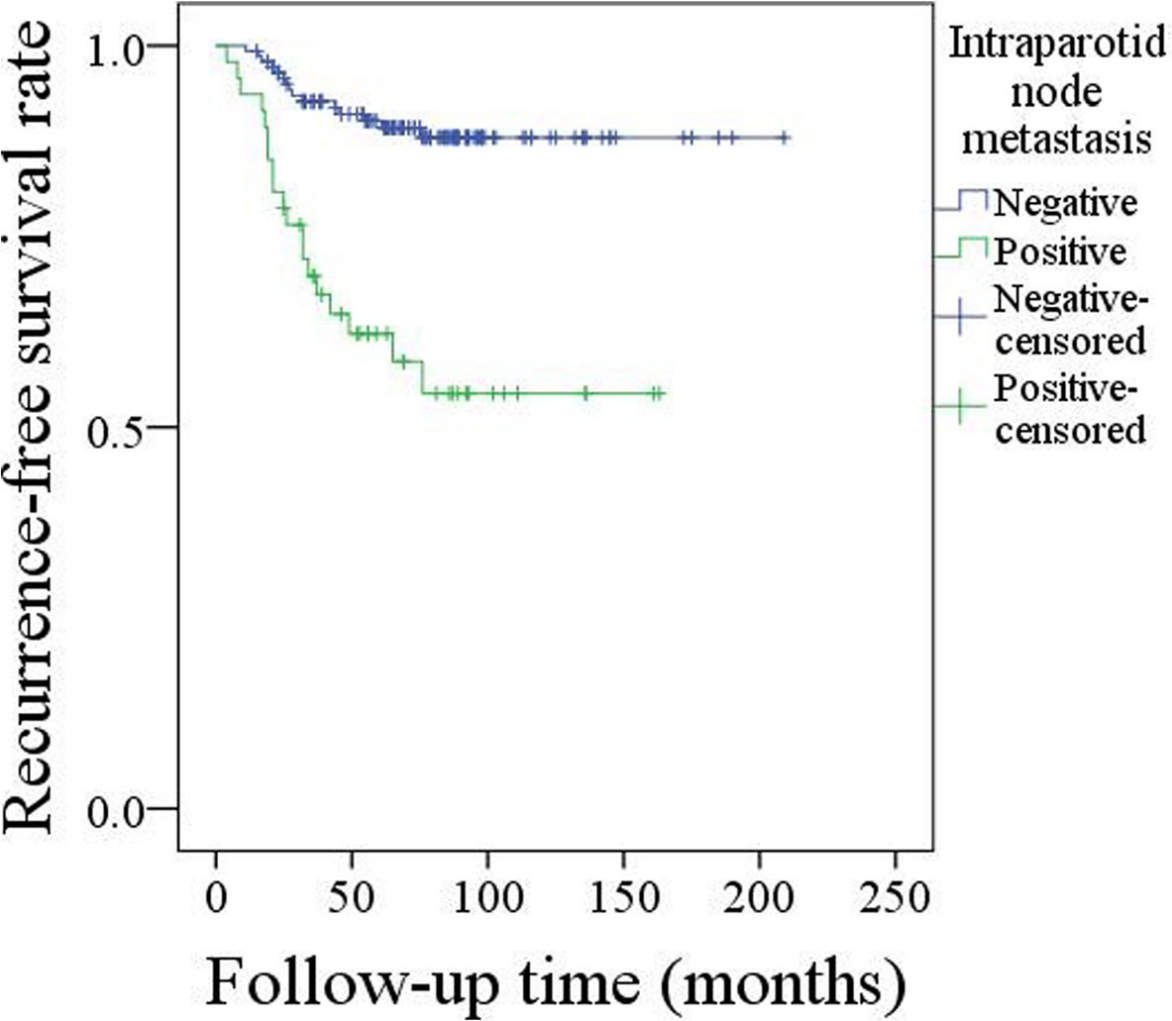
Recurrence-free survival rate in patients with or without intraparotid node metastasis (p < 0.001)

After a mean time of 71.1 months follow-up, adjuvant radiotherapy was performed in 65 patients, and recurrence occurred in 34 patients: 17 patients had local recurrence, 10 patients had neck recurrence, and 7 patients had both local and regional recurrence. The overall 10-year RFS rate was 79%. In univariable analysis, tumor stage, IPN metastasis, perineural invasion, and lymphovascular invasion as well as disease grade were significantly associated with the recurrence (Table 2, all *p* < 0.05). The 10-year RFS rate was 54% for patients with IPN metastasis, and 88% for patients without IPN metastasis, the difference was significant (*p* < 0.001) (Fig. 1). In Cox model analysis, IPN metastasis, lymphovascular invasion, and disease grade were independent prognostic factors for the RFS (Table 3, all *p* < 0.05).

**Table 2:** Univariate analysis for recurrence in patients with primary parotid cancer

Variables	p
Age (≤50 vs >50)	0.520
Sex (Female vs male)	0.116
Resection status (R0 vs R1/2)	0.384
Tumor stage (T1 + T2 vs T3 + T4)	0.004
Neck disease	0.213
Lymphovascular invasion	< 0.001
Perineural invasion	< 0.001
Disease grade	< 0.001
IPN* metastasis	< 0.001
Radiotherapy	0.495

* IPN: intraparotid node

**Table 3:** Cox model for recurrence in patients with primary parotid cancers

Variables	Exp(B)	95% CI	p
Tumor stage	1.470	0.646-3.344	0.359
Lymphovascular invasion	9.576	4.375–20.959	< 0.001
Perineural invasion	0.982	0.370–2.605	0.970
Disease grade			
Low			
Intermediate	2.592	0.953–7.048	0.062
High	3.478	1.120–10.798	0.031
IPN* metastasis	2.357	1.163–4.776	0.017

*: IPN: intraparotid node

## Discussion

Literature regarding the prognostic value of IPN metastasis was very few [8–11, 13–16]. Previous authors had reported the IPN metastasis rate ranged from 15 to 38%, and the IPN metastasis was significantly related to TNM stage and disease grade [8–11, 13–18], the finding was consistent with ours.

The IPN metastasis might affect the prognosis by two aspects. Firstly, lymph nodes existed in both lobes of the parotid gland, undiagnosed metastatic IPNs might be caused by non-total parotidectomy, and then recurrent disease might be expected owing to the residual disease. Therefore, total parotidectomy was suggested for all parotid cancer patients by previous authors [15]. The high 10-year RFS rate in current study came into our attention, it was better than previous researches [8, 11], the favorable outcome might be partially explained by our routine total parotidectomy. Another potential explanation was that high grade disease only accounted for less than 15% in our sample, and previous authors had described the disease grade was an important prognostic factor in parotid cancers [17].

Secondly, owing to the lymphatic system in head and neck area, the IPN might acted as sentinel role for finding early neck metastasis. Although Klussmann et al. [8] described pathologic neck metastasis was detected in only 33.3% of the patients with positive IPNs, both Lim et al. [12] and Nisa et al. [13] reported there was relatively high reliability of IPN metastasis in predicting neck metastasis. The viewpoint was also supported by our finding.

Risk factors for recurrence in parotid cancers had been frequently analyzed. Reported predictors included higher TNM stage, perineural invasion, lymphovascular invasion, high disease grade [13–22]. But the value of IPN metastasis remained unclear. Lim et al. [10] reported locoregional recurrence was more likely to occur in patients with cN0 neck but IPN metastasis than patients without IPN metastasis. Klussmann et al. [8] presented there was additional risk associated with the IPN involvement for tumor recurrence. Nisa et al. [11] described patients with IPN metastasis had poorer disease control. However, the three studies were limited to small sample size, and also a considerable number of patients had not undergone total parotidectomy, there might be undiagnosed IPNs. In current study, all patients underwent an total parotidectomy, it made our result more accurate, our results presented IPN metastasis was related to worse disease control, all the findings revealed that IPN metastasis carried higher risk of recurrence [23].

Association between adjuvant radiotherapy and survival benefit had been widely evaluated. In a large National Cancer Database study, Safdieh et al. [24] reported adjuvant radiotherapy improved overall survival in 4068 patients with salivary gland cancer. Similar finding was also described by Lee et al. [25]. But in current study, we failed to note there is positive relationship between radiotherapy and improved RFS. In another paper by Erovic et al. [26], the authors also presented that adjuvant radiotherapy did not significantly increase disease control in parotid cancers. More studies were required to assess the role of radiotherapy on predicting the survival in parotid cancers.

## Conclusions

In summary, the overall IPN metastasis rate in parotid MEC was 24.7%, it was significantly affected by tumor stage and disease grade. Moreover, the IPN metastasis was independently related to two-fold risk for recurrence, more adjuvant intervention was needed if there was IPN metastasis.

## Abbreviations

IPN: intraparotid node
MEC: mucoepidermoid carcinoma
RFS: recurrence-free survival
